# Magnetically boosted 1D photoactive microswarm for COVID-19 face mask disruption

**DOI:** 10.1038/s41467-023-36650-6

**Published:** 2023-02-20

**Authors:** Jeonghyo Kim, Carmen C. Mayorga-Martinez, Martin Pumera

**Affiliations:** 1 Center for Advanced Functional Nanorobots, Department of Inorganic Chemistry, Faculty of Chemical Technology, University of Chemistry and Technology Prague, Technická 5, 166 28 Prague, Czech Republic; 2 Faculty of Electrical Engineering and Computer Science, VSB—Technical University of Ostrava, 17. listopadu 2172/15, 708 00 Ostrava, Czech Republic; 3 Department of Chemical and Biomolecular Engineering, Yonsei University, 50 Yonsei-ro, Seodaemun-gu, Seoul, 03722 Korea; 4 Department of Medical Research, China Medical University Hospital, China Medical University, No. 91 Hsueh-Shih Road, Taichung, 40402 Taiwan

**Keywords:** Organic molecules in materials science, Molecular machines and motors, Synthesis and processing

## Abstract

The recent COVID-19 pandemic has resulted in the massive discard of pandemic-related plastic wastes, causing serious ecological harm and a high societal burden. Most single-use face masks are made of synthetic plastics, thus their careless disposal poses a direct threat to wildlife as well as potential ecotoxicological effects in the form of microplastics. Here, we introduce a 1D magnetic photoactive microswarm capable of actively navigating, adhering to, and accelerating the degradation of the polypropylene microfiber of COVID-19 face masks. 1D microrobots comprise an anisotropic magnetic core (Fe_3_O_4_) and photocatalytic shell (Bi_2_O_3_/Ag), which enable wireless magnetic maneuvering and visible-light photocatalysis. The actuation of a programmed rotating magnetic field triggers a fish schooling-like 1D microswarm that allows active interfacial interactions with the microfiber network. The follow-up light illumination accelerates the disruption of the polypropylene microfiber through the photo-oxidative process as corroborated by morphological, compositional, and structural analyses. The active magnetic photocatalyst microswarm suggests an intriguing microrobotic solution to treat various plastic wastes and other environmental pollutants.

## Abstract (web summary)

Most single-use face masks are made of synthetic plastics, thus their disposal poses a direct threat to wildlife as well as potential ecotoxicological effects in the form of microplastics. Here, the authors introduce a 1D magnetic photoactive microswarm capable of actively navigating, adhering to, and accelerating the degradation of the polypropylene microfiber of single-use face masks.

## Introduction

The recent COVID-19 pandemic has had a devastating impact on public health and the economy; now it threatens the global environment and natural ecosystems^1–7^. As the easiest and most effective way to slow the spread of the virus, the use of personal protective equipment (PPE), such as face masks, gloves, face shields, etc., was highly encouraged during the whole pandemic time^1,2,8^. This resulted in the massive discard of pandemic-related mismanaged plastic wastes, estimated at more than 8 million tons worldwide with >25 thousand tons discharged into the world’s oceans^3^. Single-use face masks are the most used of PPE and their monthly consumption is estimated to be 129 billion face masks globally^4^. Even only 1% of their mismanaged disposal could be equivalent to tens of tons of plastic waste released to the environment^7^. Most single-use face masks, such as surgical masks or FFP-2/N95-grade respirators, are often made of synthetic plastics such as polypropylene (PP), polyethylene (PE), or other polymeric derivatives^1,8^. These substances not only directly affect wildlife and marine organisms but also cause serious ecotoxicological effects as they gradually degrade into smaller plastic fragments, termed “microplastics”. Several studies have reported the release of plastic fibers or microplastics from disposable face masks^2,6,9^. Microplastics can be transported to waterways or oceans and ingested by marine organisms and, eventually, humans through the food chain^1,5^. In particular, recent reports have evidenced microplastics in human blood and human placenta, raising concern for their unknown interaction in the bloodstream and translocation through the blood–brain or placental barriers^10,11^. Because (i) it is difficult to fully depolymerize microplastics in natural conditions and (ii) plastic waste treatment capacity is not keeping up with the rapidly increasing rate of discharge globally, microplastics in the environment have become a serious societal problem and long-lasting threat to ecology and public health. Therefore, there is an urgent need for nanotechnological solutions to address the global plastic waste crisis.

Micro/nanorobots are at the forefront of next-generation intelligent machinery and provide intriguing strategies in various technological challenges, including therapeutics^12,13^, biofilm eradication^14,15^, environmental remediation^16–19^, and microplastic treatment^20–26^. Among these, light-driven micro/nanorobots can move under light illumination and simultaneously perform light-triggered activity; thus, they are widely used for targeted therapy^12,13^, toxin degradation^27^, and other applications^28^. However, their relatively slow speed and limitation in directional guidance are the main drawbacks that limit their usability. Also, many light-driven motions involve the use of toxic chemicals (i.e., H_2_O_2_, etc.), which hinders their applicability for biological purposes. In this regard, combining magnetic and photoactive materials in microrobots has been a fascinating design that could provide (i) fuel-free robust mechanical actuation with higher speed and force output^14,29^ and (ii) wireless and precise motion control in 2D/3D spaces^18,29,30^, integrating with light-triggered activity. Progress in the advantages of magnetic–photoactive hybrid micro/nanorobots has been demonstrated for enhanced efficiency in oral biofilm destruction^14^ and the degradation of nerve agents^18^ and organic pollutants^19,30^; however, magnetic-powered photoactive microswarms for solid waste disruption have not yet been fully explored.

**Fig. 1:**
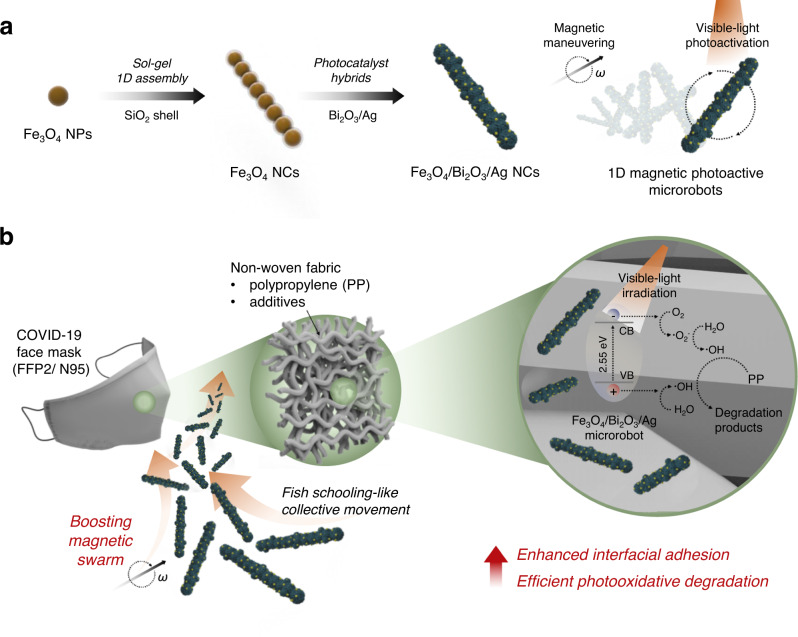
Schematic illustration of a magnetically boosted 1D photoactive microswarm for accelerated COVID-19 face mask disruption. **a** Fabrication of the hierarchically hybridized 1D magnetic (Fe_3_O_4_ NC core) and photoactive (Bi_2_O_3_/Ag shell) microrobots. **b** Programmed magnetic swarm boosting for enhanced mechanical adhesion to the PP microfiber network and sequential photoactivated disruption.

Here, we present a magnetically boosted 1D photoactive microswarm capable of actively navigating, adhering to, and accelerating the degradation of the polypropylene (PP) microfiber of COVID-19 face masks (see Fig. 1). As a unit machine of swarming, hierarchically structured 1D microrobots were fabricated that consist of the anisotropic magnetic core (Fe_3_O_4_) and photocatalytic shell (Bi_2_O_3_/Ag), thus enabling precise magnetic maneuvering and photo-oxidative activity under visible-light illumination as illustrated in Fig. 1a. As a motile backbone, the Fe_3_O_4_ nanochains (NCs) were fabricated by the magnetic field-assisted sol-gel process, in which a thin layer of silica (SiO_2_) template fixates the colloidal 1D alignments, thus providing free-standing characteristics. Their anisotropic body shape could provide more effective propulsion and higher velocity compared to the isotropic nanoparticle (NP) structure^31–33^. The 1D microrobots perform a collective tumbling motion synchronized with exerted transversal rotating magnetic fields in either manual or automated navigation mode. Also, Bi_2_O_3_ is known as a promising visible-light photocatalyst with a p-type semiconducting property and narrow band gap (2.5–2.8 eV)^34,35^; concomitantly, their heterogeneous combination with Ag could improve the efficiency of visible-light photocatalysis^36,37^. Upon the actuation of a programmed rotating magnetic field, 1D microswarms swim in synchronized speed and direction, thus exhibiting fish schooling-like collective migration inside the PP microfiber network of the COVID-19 face mask. Magnetic boosting enables active interfacial interactions, which enhance the adhesion of 1D microrobots on the PP microfiber network. The follow-up light illumination of adhered 1D microrobots generates highly oxidative species that accelerate the disruption of PP filter membranes as corroborated by morphological, compositional, and structural analyses and quantifications (Fig. 1b). Overall, the feasibility and efficacy of the magnetic photoactive 1D microswarm in the accelerated disruption of fibrous PP filter membrane are verified in a proof-of-concept demonstration with a commercial COVID-19 face mask, suggesting an intriguing microrobotic solution to treat various post-consumer plastic wastes.

## Results

### Fabrication and characterization of 1D magnetic photoactive microrobots

**Fig. 2:**
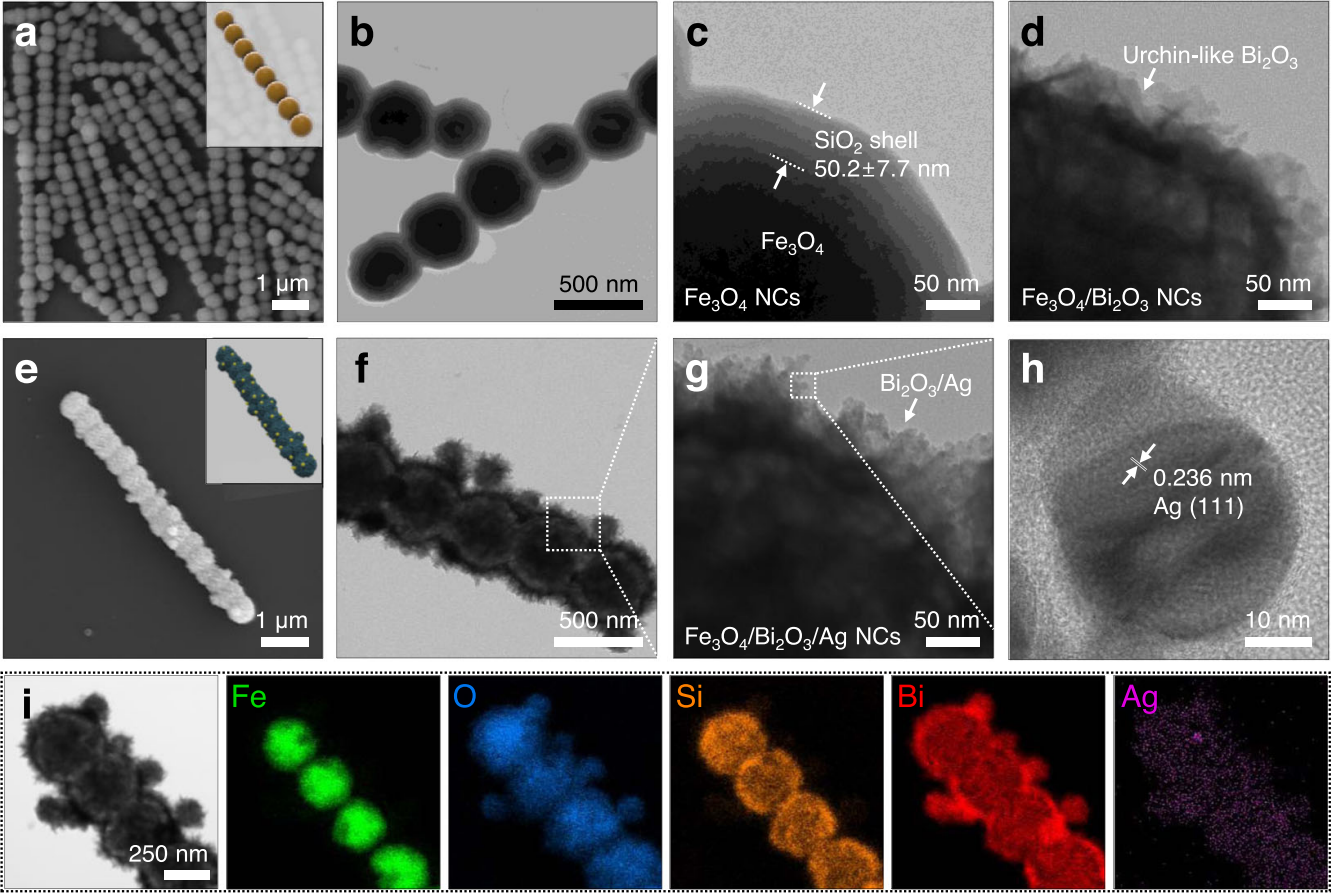
Electron microscopic characterization of the 1D magnetic photoactive microrobots. **a**, **b** Representative SEM (**a**) and TEM (**b**) images of 1D Fe_3_O_4_ NCs. **c**, **d** Magnified TEM images of Fe_3_O_4_ NC surface (**c**) and after the Bi_2_O_3_ coating (**d**). **e**–**h** Representative SEM image (**e**) and different magnification TEM (**f**, **g**), and HRTEM (**h**) images of Fe_3_O_4_/Bi_2_O_3_/Ag NCs. **i** TEM image and corresponding EDX elemental mapping images of Fe, O, Si, Bi, and Ag elements.

The procedure for fabricating the hierarchically structured 1D magnetic photoactive microrobots is illustrated in Fig. 1a. First, as a magnetic building block, colloidal Fe_3_O_4_ NPs were synthesized by the solvothermal method^38,39^. Then, the anisotropic 1D Fe_3_O_4_ colloidal NCs were assembled based on the magnetic field-assisted sol-gel process and used as the motile backbone of the microrobots^18^. Briefly, a thin layer of silica was formed on the primary Fe_3_O_4_ NPs at the early stage of tetraethyl orthosilicate (TEOS) hydrolysis. The NPs were then temporarily assembled into 1D alignment along with the applied external magnetic field. Further hydrolysis leads to the overcoating of SiO_2_, forming a rigid outer shell on the colloidal 1D alignments. Consequently, silica-templated free-standing 1D Fe_3_O_4_ NCs were obtained. Scanning electron microscopy (SEM), transmission electron microscopy (TEM), and energy-dispersive X-ray spectroscopy (EDX) analysis show that the obtained NCs have a linear colloidal alignment of Fe_3_O_4_ NPs with a smooth outer SiO_2_ shell (Fig. 2a, b and Supplementary Fig. 1). The average thickness of the silica layer was measured as 50.2 ± 7.7 nm according to 100 objectives in TEM images (Fig. 2c and Supplementary Fig. 2a).

Next, the photoactive Bi_2_O_3_/Ag hybrid shell was further coated on the Fe_3_O_4_ NC surface using subsequent solvothermal and chemical reduction processes^40–43^. In the solvothermal synthesis, Bi_2_O_3_ NPs were first hydrolyzed on the NC surface and preferentially grown to an interconnected sheet-like structure, leading to the formation of an urchin-like Bi_2_O_3_ with a rough and high surface area^40,41^. TEM and EDX mapping images reveal an urchin-like morphology of Bi_2_O_3_ that covers the entire 1D nanochain to form Fe_3_O_4_/Bi_2_O_3_ core–shell hybrids (Fig. 2d and Supplementary Fig. 1). Finally, ~20 nm Ag NPs were uniformly deposited on the rough surface of Bi_2_O_3_ by Ag precursor reduction with a strong reducing agent (NaBH_4_)^42,43^, then the NC surface was modified with the cationic polymer, i.e., poly-(diallyldimethyl ammonium chloride) (PDDA), to stabilize in positive surface charge (Fig. 2e–g)^44,45^.

The lattice fringe observed in a high-resolution TEM (HRTEM) image shows the interplanar spacing of 0.236 nm, which well corresponds to the interplanar distance of (111) planes of Ag (Fig. 2h)^46^. The resultant 1D microrobots, i.e., Fe_3_O_4_/Bi_2_O_3_/Ag NCs, have an average chain length and width of 6.9 ± 1.4 μm and 0.56 ± 0.07 μm, respectively (Supplementary Fig. 2b). The EDX mapping and spectrum further confirmed an elemental Fe is confined in the central core of linear alignment while Si, Bi, and Ag are hierarchically distributed over the whole cross-section of the core–shell NCs (Fig. 2i and Supplementary Fig. 1).

**Fig. 3:**
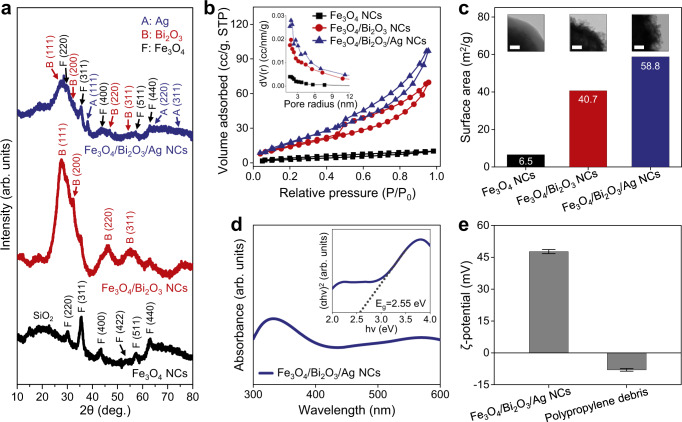
Compositional, surface, and optical properties of 1D magnetic photoactive microrobots. **a** XRD patterns of the Fe_3_O_4_ NCs (black), Fe_3_O_4_/Bi_2_O_3_ NCs (red), and Fe_3_O_4_/Bi_2_O_3_/Ag NCs (blue). **b**, **c** N_2_ adsorption–desorption isotherms (**b**), pore size distribution (inset), and comparison of surface area (**c**) for Fe_3_O_4_ NCs (black), Fe_3_O_4_/Bi_2_O_3_ NCs (red), and Fe_3_O_4_/Bi_2_O_3_/Ag NCs (blue). The inset TEM images show the magnified surface morphology of corresponding NCs. Scale bars are 50 nm. **d** UV–Vis absorption spectrum and Tauc plot (inset) of Fe_3_O_4_/Bi_2_O_3_/Ag NCs. **e** Zeta potentials of the Fe_3_O_4_/Bi_2_O_3_/Ag NCs and polypropylene debris. Error bars in **e** indicate standard deviation from triplicate measurements.

The crystallographic characteristic of the hierarchically structured Fe_3_O_4_/Bi_2_O_3_/Ag NCs is examined by X-ray diffraction (XRD) analysis (Fig. 3a). The XRD pattern of Fe_3_O_4_ NCs can be assigned to the face-centered cubic (FCC) phase of Fe_3_O_4_ (ICDD card no. 19-0629)^47,48^, but the diminished diffraction peaks and a broad peak appearing at 2*θ* = 15–25° are ascribed to the amorphous SiO_2_ shell^18,19^. After the coating of Bi_2_O_3_, additional diffraction peaks are observed that correspond to the (111), (200), (220), and (311) planes of δ-Bi_2_O_3_ crystal with a cubic phase (ICDD card no. 27-0052)^40,49^. Finally, the XRD pattern of Fe_3_O_4_/Bi_2_O_3_/Ag NCs exhibited both crystal phases of magnetite and bismuth oxide, and further characteristic peaks corresponding to the (111), (220), and (311) planes of the cubic crystal Ag phase (ICCD card no. 04-0783)^50,51^, thus confirming a composition of hybridized 1D microrobots.

To further evaluate the surface properties and roughness of the 1D microrobots, nitrogen adsorption measurements were performed. The isotherms of Fe_3_O_4_ NCs suggest non-porous surface morphology due to the smooth SiO_2_ shell, whereas after coating the urchin-like Bi_2_O_3_ shell and Ag NPs deposition, isotherms show an obvious hysteresis loop, indicating mesoporous characteristics (Fig. 3b)^50^. The Brunauer–Emmett–Teller (BET) surface area of initial Fe_3_O_4_ NCs is measured as 6.5 m^2^ g^−1^ while the Fe_3_O_4_/Bi_2_O_3_ NCs and the Fe_3_O_4_/Bi_2_O_3_/Ag NCs have a 6.3-fold (40.7 m^2^ g^−1^) and 9-fold (58.8 m^2^ g^−1^) larger surface area, respectively (Fig. 3c). Following this trend, the total pore volume for Fe_3_O_4_ NCs is 0.01269 cc g^−1^, which increased to 0.09702 cc g^−1^ and 0.13744 cc g^−1^ after coating of Bi_2_O_3_ and Ag, respectively (Supplementary Fig. 3). The dramatic increases in surface area and pore volume corroborate the rough morphology of the Bi_2_O_3_/Ag shell as observed in the TEM images, which is favorable for improving the catalytic efficiency of 1D microrobots^40,52^.

Moreover, Bi_2_O_3_ is known for its photocatalytic activity in visible-light wavelengths with a narrow band gap (2.5–2.8 eV)^34,35^ and its heterogeneous combination with Ag could improve visible-light absorption efficiency and lengthen the lifetime of photoactivated electron/hole pairs; thus, it could be a potent photoactive hybrid for efficient visible-light photocatalysis^36,37^. The ultraviolet–visible (UV–Vis) spectrum of Fe_3_O_4_/Bi_2_O_3_/Ag NCs exhibits a broad absorption profile in visible wavelengths and the band gap (*E*g) calculated from the corresponding Tauc plot corresponds to ~2.55 eV (Fig. 3d). This value is in agreement with previous reports^34,35^, implying the visible-light responsive photoactive property of the 1D microrobots.

The zeta (*ζ*) potential data in Fig. 3e show the surface charge properties of the 1D microrobots and PP debris. Interestingly, the *ζ*-potential of the PP surface is negative over a broad pH range due to the accumulated hydroxide ions in the Stern layer of polymer interfaces^53–55^. Thus, the surface charges of PP fibers and microdebris are possibly negative in aqueous or environmental (e.g., marine) pH conditions. Consistent with this trend, PP microdebris from the COVID-19 face mask shows a weak negative surface charge of −8.0 ± 0.6 mV in our measurements. Hence, the Fe_3_O_4_/Bi_2_O_3_/Ag NCs were modified with a positively charged polymer, i.e., PDDA, to provide an opposite electrostatic property^44,45^. The initial Fe_3_O_4_ NCs have a negative surface charge (*ζ* = −29.7 ± 0.4 mV) due to the abundant hydroxyl terminal groups on the SiO_2_ template layer, and the coating of the Bi_2_O_3_/Ag shell increase the *ζ*-potential to −7.8 ± 0.7 mV (Supplementary Fig. 4). After the PDDA treatment, the final Fe_3_O_4_/Bi_2_O_3_/Ag NCs had a positive surface charge with a ζ-potential value of +47.7 ± 1.0 mV.

### Magnetic-powered motion control and self-navigating 1D magnetic photoactive microrobots

The 1D magnetic photoactive microrobots were wirelessly actuated by transversal rotating magnetic fields (further details on the rotating magnetic field generation system are given in the Experimental Section)^42,56^. The 1D microrobots are propelled with a continuous vertical tumbling motion along the exerted rotating magnetic fields in the *x*–*z* plane. Also, controlling of the rotational plane angle in the *x*–*y* plane enables directional control when operated in either manual or automated navigation mode. In this condition, the anisotropic shape of Fe_3_O_4_ NCs is advantageous compared to the isotropic NP structure, as it could provide more robust propulsion behavior in microtopographies due to the smaller flow fields and resistive forces^31^. Besides, the magnetic moment is higher in chain structure than single NP, allowing a stronger magnetic response, and thus the increase of propulsion velocity^32,33^.

**Fig. 4:**
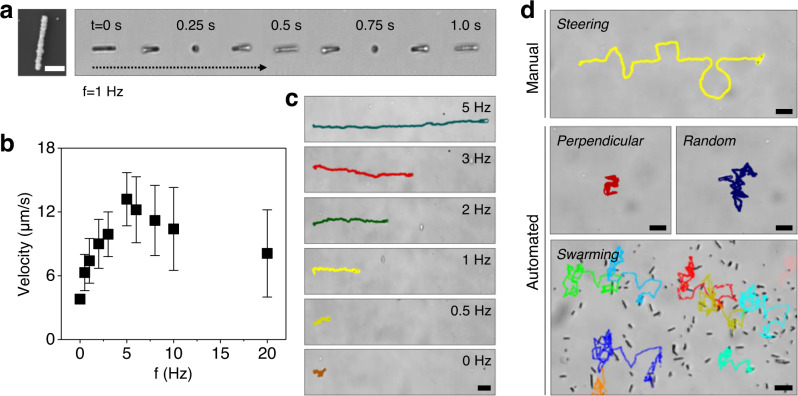
Magnetic-powered propulsion, speed, and various locomotion modes of 1D magnetic photoactive microrobots. **a** Time-lapse images of 1D microrobot propulsion (right) and an SEM image of the 1D microrobot (left). Scale bar is 2 µm. **b**, **c** Average velocities of the 1D microrobots as a function of rotating magnetic field frequency (**b**) and representative propulsion trajectories (**c**). Scale bar is 10 µm. **d** Manual and automated locomotion modes and magnetic swarm boosting for programmed navigation (5 mT, 5 Hz). Scale bars are 10 μm. Data in **b** are presented as mean ± standard deviation (*n* = 15).

Figure 4a shows time-lapse images of typical forward propulsion under a 5 mT and 1 Hz rotating magnetic field in which the completion of a single tumbling cycle takes 1.0 s; this implies that the rotating motion of 1D microrobots is synchronized with the input rotational frequency of the magnetic field. Since the tumbling motion is translated to forward movement, the propulsion velocity is linearly increased with applied rotating frequencies from 0.5 to 5 Hz as shown in Fig. 4b, c. However, when increasing the input frequency further, the magnetic torque is insufficient to keep synchronized rotation and the liquid-induced viscous torque becomes much high, which causes the decrease of rotational and propulsion velocities of the 1D microrobots^57^. Therefore, the maximum velocity can be achieved at the highest synchronized frequency, which is called the step-out frequency. The 1D microrobots reach their maximum velocity of 13.2 µm s^−1^ with a step-out frequency of 5 Hz. Beyond this frequency, the 1D microrobots become unsynchronized and begin to lower their average velocities. A real-time comparison of 1D microrobots propelled via different frequencies is presented in Supplementary Movie 1.

Besides, the magnetic controller used to manipulate the rotational plane angle provides diverse steering options. Figure 4d demonstrates various locomotion modes and trajectories that can be operated in manual or automated fashion. In the manual navigation mode, controlling the rotation angle leads to steerable locomotion of the 1D microrobots (Fig. 4d, image in the top row, and Supplementary Movie 2). In the automated navigation mode, the 1D microrobots propel along a given trajectory with predefined rotation angle generation; for example, in the continuous cycle of perpendicular or random directional locomotion (Fig. 4d, images in the middle row, and Supplementary Movie 3)^42,56^. In addition, magnetically boosted collective motion is demonstrated in Fig. 4d (images in the bottom row) and Supplementary Movie 4, showing that the automatically navigating 1D microswarm following the programmed trajectory in random locomotion mode. Collective activation of dense magnetic microrobot swarms commonly suffers from uncontrollable aggregation due to the magnetic dipole attraction^33^. In our work, the 1D microrobots are composed of superparamagnetic Fe_3_O_4_ NPs with negligible remanence^38^ and possess sufficient colloidal stability, both minimizing the disordered aggregation in large number operation. Thus, the 1D microswarms swim in synchronized speed and direction, simultaneously forming internal collective order by communicating between individuals to balance attraction and repulsion. This programmed swarming with repeated forward and backward motion in planned trajectories enables the powering of self-navigating 1D microrobots that continuously interact with environmental obstacles (e.g., microfiber matrix, etc.). Also, the 1D microrobots exhibit an instantaneous response to switching of mobile speed and directionality in a negligible inertial force environment^57,58^, thus enabling accurate navigation and maneuvering of collective movements on demand. Taken together, the magnetically boosted 1D microswarm demonstrated an untethered control in multiple locomotion modes, therefore implying its use as an active and intelligent microscale collective for further practical applications.

### Magnetically boosted 1D microswarm and enhanced adhesion to the polypropylene microfiber membrane

The fabricated 1D microrobots are capable of collective magnetic maneuvering and simultaneously possess photocatalytic activity in visible-light wavelength. Thus, they were especially envisioned to perform an intriguing strategy for challenging tasks, i.e., microrobotic swarm-based photoactive disruption of disposable COVID-19 face mask that is the pandemic-related plastic waste causing the environmental threat. As a proof-of-concept demonstration, the most commonly used commercial FFP-2-grade face mask was chosen as a model pollutant. Single-use face masks, such as surgical masks or FFP-2/N95-grade respirators, consist of three or four layers of melt-blown nonwoven fibrous membranes that are often made of PP synthetic plastics^1,8^. Different from the flat surface of plastic products, these fibrous polymeric membranes comprise entangled PP microfibers with a diameter of ≈20 μm and micrometer-scale voids between the entangled microfiber network^8^. It is, therefore, more difficult for the photocatalysts to access the microfiber surface uniformly. In this regard, magnetic actuation suits because the actuating fields can penetrate the depth of a 3D microfiber network and wirelessly control the movements of the 1D microswarm. Moreover, subsequent visible-light-driven photocatalytic activation can accelerate the decomposition of the PP polymeric filter membranes, which is estimated to take as long as hundreds of years in a natural environment.

**Fig. 5:**
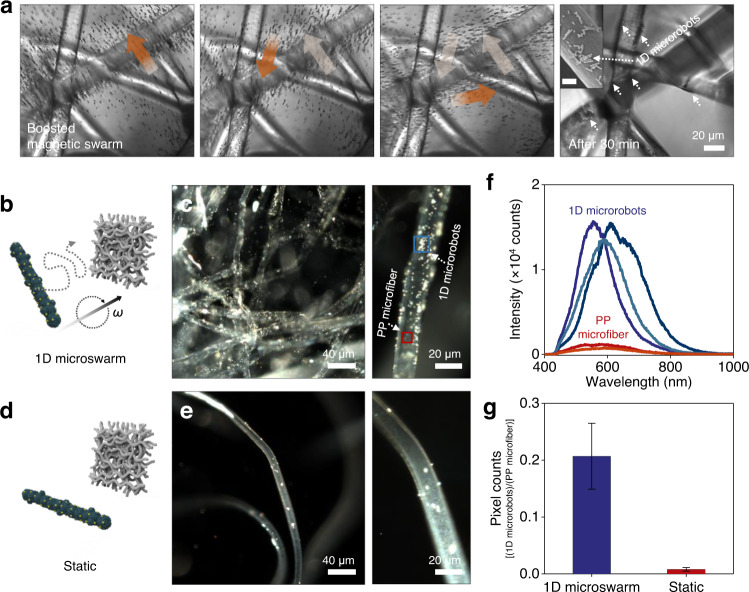
Fish schooling-like movement of 1D microswarm and enhanced adhesion on the microfiber network. **a** Time-lapse microscopy images illustrating the magnetically boosted 1D microswarm and their robust interaction with the PP microfiber network (see also Supplementary Movie 5). The white arrows in the last image and inset SEM image highlight the adhered 1D microrobots on the microfiber network after 30 min actuation (Scale bar in the inset is 5 μm). **b**–**g** Hyperspectral dark-field microscopy (HDFM) analysis for the quantification of the adhered 1D microrobots on the PP microfiber network. Schematic illustrations (**b**, **d**) and enhanced dark-field microscopy images (**c**, **e**) of the magnetically boosted 1D microswarm (**b**, **c**) or static 1D microrobot (**d**, **e**)-treated microfiber samples. Spectral profiles collected from several pixels of 1D microrobots and PP microfiber network (**f**), and the comparison of pixel count ratio factors between the 1D microswarm and static 1D microrobot-treated PP membrane groups (**g**). Error bars in (**g**) denote standard deviation from triplicate measurements.

To explore the dynamic interaction of the 1D microswarm with the interfaces of the microfibers, small pieces of PP filter membranes were prepared and dipped into the 1D microrobots dispersion, and then placed inside the electromagnetic coil setup (see details in the Experimental Section). Supplementary Movie 5 and the corresponding time-lapse images in Fig. 5a demonstrate the magnetically boosted 1D microswarm interacting with the PP microfiber network. Hundreds of 1D microrobots were deployed and swam in synchronized speed and direction along the actuated rotating magnetic fields (5 mT, 3 Hz), thus exhibiting fish schooling-like collective movements with continuous random directional navigation. This cooperative behavior achieves the collective’s migration alongside and across the PP microfiber network repeatedly, leading to active interfacial interaction and enhanced physical contact time. As the 1D microrobots were designed to have an opposite electrostatic property to the PP microfiber as described above, they could be adhered to the microfiber surface by either physical or electrostatic attraction (Fig. 3e). After continuous microswarm boosting for 30 min, a large amount of 1D microrobots remained on the microfiber network as shown in the last frame in Fig. 5a and Supplementary Movie 5. Conversely, in the absence of magnetic actuation, the static 1D microrobots had passive interaction with the microfiber network, which leads to much less surface adhesion compared to the active 1D microswarm (Supplementary Movie 6).

Hyperspectral dark-field microscopy (HDFM) with spectral mapping technique helped us quantitatively analyze the efficiency of 1D microrobots’ adhesion on the PP microfiber network (Fig. 5b–g and Supplementary Fig. 5). Two groups of PP filter membranes were prepared and treated with magnetically boosted 1D microswarm or static 1D microrobots for 30 min as schematically illustrated in Fig. 5b, d (see details in the Experimental Section). The enhanced dark-field microscope images showed apparent differences between the two groups; in the case of the 1D microswarm-treated filter membrane, the PP microfiber network was densely covered with the 1D microrobots as indicated by shiny dots due to the strong scattering of Fe_3_O_4_/Bi_2_O_3_/Ag NCs (Fig. 5c). On the contrary, the static 1D microrobots passively interacted with the filter membrane; hence, the shiny dots are sparsely located on the PP microfiber network (Fig. 5e).

Figure 5f illustrates the spectral profiles collected from several different pixels corresponding to the 1D microrobots (shiny dots) or the PP microfibers (dimmed gray fibers). The spectral profiles from the 1D microrobots show a distinct scattering peak in wavelengths around 600 nm, which obviously differs from the PP microfiber background spectra with a highly reduced scattering intensity. The 1D microrobots on the microfibers could be quantified using this significant difference in their spectral intensities. By exploiting the particle filter algorithm and spectral mapping, the pixel areas indicating 1D microrobots or microfiber backgrounds were separately mapped and counted in the hyperspectral images as illustrated in Supplementary Fig. 5. The relative quantity of adhered 1D microrobots could be defined in terms of pixel count ratio factor, i.e., the ratio of pixel counts indicating 1D microrobots and microfiber background area (Eq. (1)):

1 $${{{{{\rm{Relative}}}}}}\,{{{{{\rm{quantity}}}}}}\,{{{{{\rm{of}}}}}}\,{{{{{\rm{adhered}}}}}}\,1{{{{{\rm{D}}}}}}\,{{{{{\rm{microrobots}}}}}}=\frac{{{{{{\rm{Pixel}}}}}}\,{{{{{\rm{counts}}}}}}\,{{{{{\rm{of}}}}}}\,1{{{{{\rm{D}}}}}}\,{{{{{\rm{microrobots}}}}}}}{{{{{{\rm{Pixel}}}}}}\,{{{{{\rm{counts}}}}}}\,{{{{{\rm{of}}}}}}\,{{{{{\rm{microfiber}}}}}}\,{{{{{\rm{network}}}}}}}$$

The pixel count ratio factor of the PP filter membrane treated with 1D microswarm was 0.207 ± 0.058, which represents *a* ≈ 26-fold increase compared with that of the static 1D microrobot-treated filter membrane (0.008 ± 0.003) and indicates the highly enhanced adhesion efficiency of the magnetically boosted 1D microswarm (Fig. 5g). As the photocatalytic oxidative species are mainly generated on the surface of photocatalysts and diffused to the surface of the target reactant^59^, large coverage of the 1D microrobots with intimate interfacial contact is essential and highly advantageous for promoting the photocatalytic oxidation of the PP filter membrane.

### Photo-oxidative effect of 1D microrobots to accelerate the disruption of polypropylene filter membranes

**Fig. 6:**
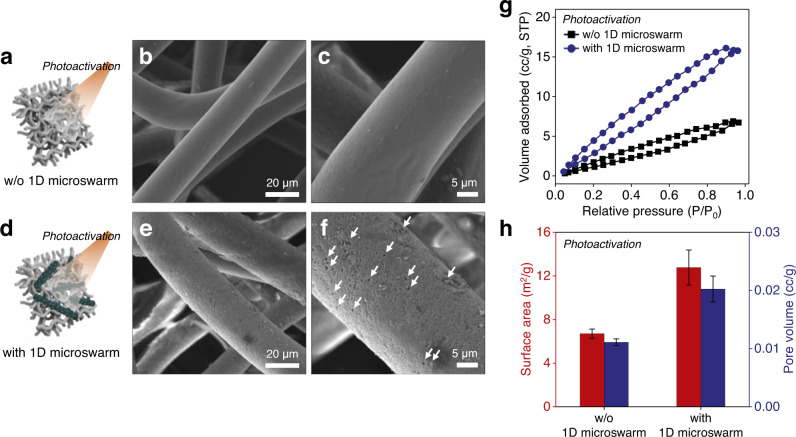
Morphological characterization of the polypropylene filter membranes after photoactivation with and without 1D microswarm. **a**–**f** Schematic illustrations and different magnification SEM images of the microfiber network after photoactivation without (**a**–**c**) or with treatment of the 1D microswarm boosting (**d**–**f**). **g**, **h** BET surface area analysis for the quantification of morphological disruption of the filter membranes. Comparison of N_2_ adsorption–desorption isotherms (**g**), surface area, and BET total pore volumes (**h**) after photoactivation with and without 1D microswarm boosting. Error bars in **h** indicate standard deviation from triplicate measurements.

After validating the enhanced coverage of 1D microrobots through robust collective motion, we proceeded to examine their photocatalytic activity to promote the disruption of the PP microfiber network. Two groups of PP filter membranes—with and without the 1D microswarm treatment—were exposed to UV-visible light illumination that mimicked solar irradiation. Figure 6 presents a comparison of morphological features of the PP filter membranes after 30 h of light illumination. The filter membrane without treatment of 1D microswarm actuation shows a smooth and uniform surface of the microfiber network, indicating that the current condition of light irradiation is not capable of inducing distinct degradation (Fig. 6a–c). Conversely, we can observe the significant differences in the 1D microswarm-treated PP filter membrane, which contains cracks and cavities with a diameter of 100–500 nm over the whole surface of the PP microfiber network (Fig. 6d–f). This result is consistent with previous observations regarding the formation of cracks and cavities, and implies that the photogenerated oxidative species from the 1D microrobot surface cause breakage of the macromolecular chains; then, their penetration deeper inside the defects of the microfiber network facilitates further photo-oxidative reaction, leading to the destruction of the polymeric matrix^60–63^.

Furthermore, the degree of physical deformation accompanying the cracks and cavities could be quantitatively characterized by BET surface area and porosity analysis. Figure 6g, h, respectively, illustrate the typical N_2_ adsorption–desorption isotherms and corresponding surface area, and the total pore volume plots of the PP filter membranes after 30 h of light illumination. The PP filter membranes without treatment of 1D microswarm show a lower BET surface area of 6.7 ± 0.4 m^2^ g^−1^ and pore volume of 0.01110 cc g^−1^ while the 1D microswarm-treated PP filter membranes exhibit an increase of ≈1.9-fold (12.8 ± 1.6 m^2^ g^−1^) in surface area and ≈1.8-fold (0.02025 cc g^−1^) in pore volume, respectively, due to the damaged rough surfaces with small cracks and cavities. These results allow us to compare the degree of morphological disruption quantitatively and the observed considerable increase in surface area and porosity confirm the high degree of deformation over the entire surface of PP filter membranes caused by the photocatalytic activity of 1D microswarm.

**Fig. 7:**
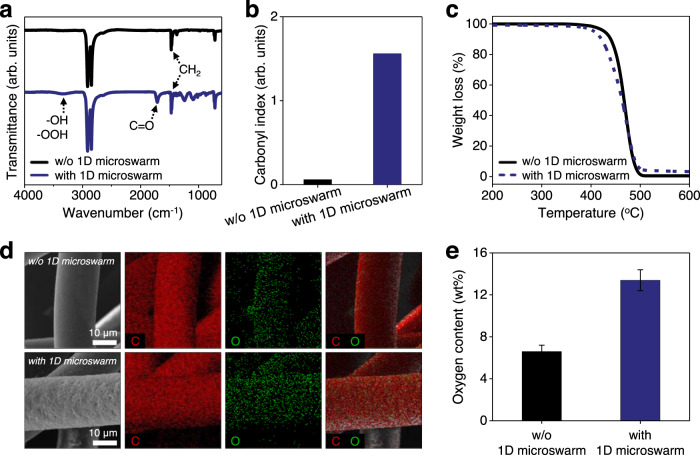
Compositional and structural characterization of the polypropylene filter membranes after photoactivation with and without 1D microswarm. **a** FTIR-ATR spectra, **b** carbonyl index, and **c** TGA curves of the PP filter membranes after photoactivation with and without treatment of the 1D microswarm boosting. **d**, **e** Representative SEM-EDX elemental mapping images after photoactivation (**d**) and comparison of oxygen content in elemental composition determined by EDX analysis (**e**). Error bars in **e** denote standard deviation from triplicate measurements.

To further evaluate the photo-oxidative effect of 1D microrobots during the disruption of the PP microfiber network, compositional and structural characterizations were performed for the PP filter membranes after the photo-oxidation reaction. First, Fourier-transform infrared spectra with attenuated total reflectance (FTIR-ATR) measurements confirmed the chemical composition changes of the photocatalyzed PP filter membranes (Fig. 7a). As photo-oxidation of the polymeric matrix could be evidenced by characteristic peaks in the infrared region, this is an effective method to investigate the state of deterioration of the PP filter membranes^60,64,65^. The FTIR-ATR spectra of both light-illuminated PP filter membranes exhibit several peaks of CH_2_ stretching and bending vibrations (2914 cm^−1^, 2846 cm^−1^, 1471 cm^−1^, and 1461 cm^−1^)^61,64,65^, representing the typical characteristics of a PP matrix. However, two distinct peaks at ~1712 cm^−1^ and ~3350 cm^−1^ appear in the spectrum of the 1D microswarm-treated PP filter membranes, indicating the formation of carbonyl (C=O) and hydroxyl/hydroperoxyl (–OH, –OOH) groups on the oxidized sites as major oxidation products^60,64,65^. The degree of photo-oxidation could be compared in terms of a carbonyl index (CI), which is a common indicator measuring carbonyl species formed during oxidation processes^60,65^. The CI was calculated by dividing the area of the carbonyl peak (C=O, 1850 cm^−1^ to 1650 cm^−1^) between the area of the methylene scissoring peak (CH_2_, 1500–1420 cm^−1^) following the previously reported method (Eq. (2))^65^:

2 $${{{{{\rm{Carbonyl}}}}}}\,{{{{{\rm{index}}}}}}\,({{{{{\rm{CI}}}}}})=\frac{{{{{{\rm{Area}}}}}}\,{{{{{\rm{under}}}}}}\,{{{{{\rm{band}}}}}}\,1850-1650\,{{{{{\rm{cm}}}}}}}{{{{{{\rm{Area}}}}}}\,{{{{{\rm{under}}}}}}\,{{{{{\rm{band}}}}}}\,1500-1420\,{{{{{\rm{cm}}}}}}}$$

As shown in Fig. 7b, the CI of the 1D microswarm-treated PP filter membranes was ≈26-fold higher than that of the PP filter membranes without treatment of 1D microswarm, confirming the notable carbonyl group formation on the oxidized sites of the filter membrane and the efficient photo-oxidative reaction caused by the 1D microswarm.

Figure 7c compares the thermal decomposition profiles of the light-illuminated PP filter membranes as examined by thermogravimetric analysis (TGA). The thermogram for the PP filter membrane without treatment of 1D microswarm presents a 10% weight loss at ~440 °C and then steep decomposition at 440–500 °C, reaching a weight loss of 99.5%. On the other hand, the thermogram of the 1D microswarm-treated PP filter membrane reached a 10% weight loss at 20 °C lower temperature (~421 °C) and major decomposition occurred at 420–500 °C, resulting in a weight loss of 96.2%. This finding is further clarified with a shoulder peak arising at a lower temperature in the DTG curve (Supplementary Fig. 6). The observed TGA curve shift to a lower temperature implies the reduced thermal stability of the 1D microswarm-treated PP filter membrane, plausibly due to the breakage of the macromolecular chains and decreased crystallinity after the photo-oxidative process, which is in agreement with observations in previous reports^60,64^.

Further, elemental composition analysis using SEM-EDX mapping and spectra reveals the different oxygen content (wt%) on the light-illuminated PP filter membranes (Fig. 7d, e). Figure 7d illustrates the typical elemental maps of the PP filter membranes after reactions. The relative elemental compositions for carbon (C) and oxygen (O) were obtained in three different sites of both light-illuminated PP filter membranes and then quantified as shown in Fig. 7e and Supplementary Fig. 7. The oxygen content on the 1D microswarm-treated PP filter membrane was measured to be 13.4 ± 1.0 wt%, indicating a prominent increase of ≈2.04-fold in mass fraction of oxygen compared to the PP filter membrane without treatment of 1D microswarm (6.6 ± 0.6 wt%). The observed higher oxygen distribution is indicative of the increased number of oxidized sites over the filter membrane after the photo-oxidative process with 1D microrobots^64^, which is consistent with the FTIR results showing the formation of carbonyl (C=O) and hydroxyl/hydroperoxyl (–OH, –OOH) groups.

Following the investigation of accelerated disruption, the liquid sample after the photocatalytic treatment of the PP filter membrane was further analyzed by solid phase microextraction coupled with gas chromatography-mass spectrometry (SPME-GC-MS), which shows that the complex organic compounds released from the PP filter membrane, including the abundant by-products of ethynyloxy/acetyl radical (*m*/*z* = 41, 43), acetaldehyde (*m*/*z* = 44), and 2-Propynyl, 1-hydroxy-(*m*/*z* = 55), as shown in Supplementary Fig. 8^60^. Furthermore, the potential cytotoxic effect of by-products in the reacted sample was evaluated by in vitro cytotoxicity assay in the HeLa cell line. After the 30 h of photocatalytic treatment for 1D microswarm-treated PP filter membranes, the resulting reaction solution was collected and incubated with the cells at different concentrations for 2 h and 72 h. The cytotoxicity was evaluated using a resazurin assay. As shown in Supplementary Fig. 9, the toxicological profiles show negligible influence on cell viability expressed as metabolic activity at all tested conditions, indicating the biological toxicity caused by photocatalytic degradation products and residual reagents is insignificant.

### Active magnetic collection of 1D microswarm-treated polypropylene filter membrane and microdebris

The active collection and removal of PP plastic wastes from the contaminated waters would be another compelling capability for the perspective in practical scenarios. We demonstrated the straightforward separation of the 1D microswarm-treated PP filter membrane and its microdebris using an external magnetic field, as shown in Supplementary Movie 7. This performance allows possible routes for practical applicability. For example, the 1D microswarm-treated PP wastes can be magnetically collected and then go through the photodegradation process, which can provide a more efficient treatment procedure. Also, the plastic fiber debris that can be fragmented upon the exposure to photocatalytic process or natural mechanical and physical factors is readily extracted from the solution for the post-treatments.

## Discussion

In summary, we have presented a 1D magnetic photoactive microswarm for active navigating and adhering to and accelerating the degradation of the polypropylene microfiber of a COVID-19 face mask. As a unit machine of swarming, the Fe_3_O_4_/Bi_2_O_3_/Ag 1D microrobots were designed to have high surface area, visible-light photocatalytic property, and positive surface charge. The 1D microrobots were actively propelled in a tumbling motion with controllable speed and direction. In particular, the automated navigation modes with continuous locomotion in planned trajectories enable the boosting of a self-navigating 1D microswarm that exhibits fish schooling-like collective migration. The magnetically boosted 1D microswarm leads to ≈26-fold enhanced adhesion on the PP microfiber network, which is evaluated by hyperspectral dark-field microscopy. After 30 h of light irradiation, physical deformation with ≈1.9-fold increased surface area, ≈26-fold higher oxidized functional groups, weakened crystallinity, and enlarged oxidized sites were evidenced, confirming the accelerated disruption of the PP filter membranes through the photo-oxidative process.

As summarized in Supplementary Table 1, current microrobot-based approaches to treat plastic wastes mainly rely on the removal of target plastics^20–23^, which need to be advanced to process that enables collection and degradation, simultaneously. In this respect, light-powered micro/nanorobots are promising as they can concurrently move and photocatalysis, but their robust activation is limited by the necessity of toxic chemicals (i.e., H_2_O_2_, etc.)^20,24,25^. In addition, recent studies are continuing efforts to demonstrate their practicality through samples derived from commercial plastic products, rather than plastic particle models^20,24–26^. From these points of view, the strategy with 1D magnetic photoactive microswarm provides several advantages for practical application to treat fibrous plastic wastes: (i) the magnetic actuation does not require the use of toxic chemicals that can cause secondary environmental pollution. Besides, the magnetic actuating fields can penetrate the depth of a 3D-structured microfiber network and wirelessly control the robust 1D microswarm in a less accessible area, which better suits than other driving forces; (ii) the high surface area of urchin-like Bi_2_O_3_ and its combination with Ag afford an efficient visible-light photocatalytic effect for accelerating the degradation of PP filter membranes; (iii) the robust swarm movement can enhance physical adhesion, allowing intimate and homogeneous coverage of photocatalysts on the microfiber network and, thus, effective oxidative reaction; and (iv) the 1D magnetic photoactive microswarm treatment is capable of integrating magnetic collection/removal and photodegradation, enabling the streamlined process with high efficiency in future practical application.

Despite the progress made in this study, the complete degradation of solid plastics is still challenging in the current process. Most photocatalytic depolymerization techniques also suffer from limited degradation and exhibit low efficiency even with a reaction time of tens to hundreds of hours^66,67^. While these processes can accelerate the degradation of synthetic plastics, which can take tens or hundreds of years in a natural environment, it should be further improved sufficiently. Additionally, the need for a magnetic manipulation setup may lead to limited scalability. Materials cost for the synthesis of microrobots also should be reduced to be affordable higher volume treatment. Therefore, future works need to ameliorate these problems by exploring highly efficient materials and designing processes more applicable to large-scale applications.

Overall, the promise of the magnetic photoactive 1D microswarm for accelerating the degradation of fibrous synthetic plastics is demonstrated using a commercial COVID-19 face mask. Active magnetic photocatalyst microswarm suggests an intriguing microrobotic solution to treat various post-consumer plastic wastes and other environmental pollutants.

## Methods

### Materials

Iron(III) chloride hexahydrate (FeCl_3_·6H_2_O), trisodium citrate dihydrate (C_6_H_5_Na_3_O_7_·2H_2_O), sodium acetate (CH_3_COONa), ammonium hydroxide solution (NH_4_OH, 28 wt%), tetraethyl orthosilicate (TEOS), bismuth nitrate pentahydrate (Bi(NO_3_)_3_·5H_2_O), silver nitrate (AgNO_3_), sodium borohydride (NaBH_4_), ethylene glycol (EG), and poly-(diallyldimethyl ammonium chloride) solution (PDDA, 20 wt% in H_2_O) were purchased from Sigma-Aldrich, USA. All aqueous solutions were prepared using Milli-Q-grade (> 18.2 mΩ/cm) deionized (DI) water.

### Synthesis of Fe_3_O_4_@Bi_2_O_3_@Ag 1D microrobots

The Fe_3_O_4_ NPs were synthesized using a solvothermal method^38,39^. In a typical synthesis, FeCl_3_·6H_2_O (0.54 g), trisodium citrate (0.2 g), and sodium acetate (1.2 g) were dissolved in 20 mL of EG, followed by vigorous stirring for 30 min. The mixture was transferred in a Teflon-lined stainless-steel autoclave, and the reaction was allowed at 200 °C for 10 h. The resultant black products were washed with ethanol and DI water three times and dried in a vacuum oven at 60 °C for 6 h. The SiO_2_-templated 1D Fe_3_O_4_ colloidal NCs were assembled by the magnetic field-assisted sol-gel process as described elsewhere^18^. Briefly, an aqueous solution of 2 mL Fe_3_O_4_ NPs (5 mg/mL) was mixed with 800 μL of NH_4_OH (28 wt%) and 16 mL of ethanol, and then dispersed well under sonication for 30 min. Into the prepared dispersion, 180 μL of TEOS was quickly injected and then kept in sonication for 15 min. Afterward, the prepared solution was exposed to a static magnetic field for 15 s and then allowed 20 min more reaction time in an undisturbed state. The resultant solution was washed with ethanol and DI water three times and dried in a vacuum oven. Next, the urchin-like Bi_2_O_3_ shell was overcoated on the 1D NC structure by a subsequent solvothermal process^40,41^. In a typical procedure, 0.194 g of Bi(NO_3_)_3_·5H_2_O (0.4 mmol) was dissolved in a mixture of ethylene glycol (17 mL) and ethanol (34 mL), then vigorously stirred for 30 min. After that, the as-prepared 1D Fe_3_O_4_ NCs (10 mg) were mixed and sonicated for 30 min, and then transferred in a Teflon-lined stainless-steel autoclave (70 mL capacity). The reaction was allowed at 160 °C for 5 h. The resultant products were washed with ethanol and DI water three times followed by drying in a vacuum oven. Finally, ~20 nm Ag NPs were deposited on the surface of Bi_2_O_3_ by the chemical reduction method^42,43^. The as-prepared 1D Fe_3_O_4_/Bi_2_O_3_ NCs (10 mL, 1 mg/mL) were mixed with 10 mL of Ag precursor-citrate solution containing 5 mM AgNO_3_ and 38.8 mM trisodium citrate. Then, 100 μL of freshly prepared 0.1 M NaBH_4_ was quickly added to the mixture under sonication. The immediate solution color change indicates the formation of Ag NPs on the NCs and the reaction is continued under sonic irradiation for 30 min. For PDDA-functionalization, an aqueous dispersion of 1 mL Fe_3_O_4_/Bi_2_O_3_/Ag NCs (0.2 mg/mL) was mixed with 500 μL of PDDA solution (1%) and allowed sonication for 1 h^44,45^. The final products were then washed with DI water three times and dried in a vacuum oven for further use.

### Characterization of Fe_3_O_4_@Bi_2_O_3_@Ag 1D microrobots

The morphology and chemical composition of the Fe_3_O_4_@Bi_2_O_3_@Ag 1D microrobots were analyzed by scanning electron microscopy (MAIA3, Tescan, Czech Republic) and transmission electron microscopy using a Jeol JEM-2200FS (Japan) equipped with an energy-dispersive X-ray spectrometer (EDX). X-ray diffraction (XRD) analysis was performed using an X-ray diffractometer (Bruker, D8-advance, Germany). Brunauer−Emmett−Teller (BET) surface area and pore volume were measured using a NOVAtouch LX2 instrument (Quantachrome Instrument, USA). UV−Vis spectra were acquired by a UV-Vis spectrophotometer (UV-2600, Shimadzu, Japan). Surface potentials were analyzed by a zeta-sizer (Zetasizer Pro, Malvern Instruments Ltd., UK).

### Magnetic-powered motion study

The motion of 1D magnetic photoactive microrobots was wirelessly controlled using a customized triaxial electromagnetic coil setup that can generate transversal rotating magnetic fields with tunable magnetic field frequencies and rotating plane angles. More details on the magnetic manipulation setup can be found in the authors’ previous reports^42,56^. The microscopic motion study of 1D microrobots and the interaction of 1D microswarm in a microfiber network were performed using an inverted optical microscope (Olympus IX73) equipped with a Basler acA-1920-155 μm monochrome CMOS camera and 50× objective lens. In a typical motion analysis, 50 μL of 1D microrobot dispersion (0.1 mg/mL) was dropped on a glass slide and then placed inside the triaxial electromagnetic coils. The average velocity and tracking trajectories were acquired from the recorded videos using NIS-Elements AR 3.2 software.

### Quantification of adhered 1D microrobots by HDFM analysis

All experiments on 1D microswarm interaction and photodegradation were carried out using commercial FFP-2-grade respirators (JW™ Respirator CNJW-2020 FFP2 NR, China). To investigate the dynamic interaction of the 1D microswarm with the microfiber surfaces, small pieces of the PP filter membranes from the COVID-19 face mask were prepared in a rectangular form (≈3 × 4 mm) and washed with DI water before use. Subsequently, the PP filter membranes were put into test tubes and fully soaked in the aqueous dispersion of 1D magnetic photoactive microrobots (0.2 mg/mL). One group of test tubes was located inside the electromagnetic coil setup and the rotating magnetic fields were applied in random locomotion mode (5 mT, 3 Hz) for 30 min (i.e., magnetic swarming group) while the other group of test tubes remained in an undisturbed state without magnetic actuation (i.e., static group). Hyperspectral dark-field microscopy (HDFM) analysis was carried out using the CytoViva Hyperspectral Imaging System (CytoViva Inc., USA). Dark-field images and hyperspectral maps were obtained using 20× and 60× objective lenses, and then processed by ENVI data analysis software (ENVI 4.8). The locations of pixels accounting for 1D microrobots or the PP microfiber network were mapped and quantified using a particle filter algorithm under the following conditions: 1D microrobots: peak wavelength in 650 ± 50 nm, peak intensity over 5000 counts; PP microfiber network: peak wavelength in 600 ± 10 nm, peak intensity over 1000 counts. The relative quantity of adhered 1D microrobots was estimated by the pixel counts ratio factor according to Eq. (1).

### Photodegradation of PP microfibers and characterizations

The test group of PP filter membranes was treated with 1D microswarm following the same procedure described above and the control group of PP filter membranes was prepared without 1D microswarm treatment. Subsequently, both groups of test vials were placed below the 300 W Ultra-Vitalux Osram lamp at a distance of 30 cm, followed by irradiation for 30 h. After the photoactivation, the PP filter membranes were washed three times with DI water and characterized. Morphological disruption was analyzed by SEM imaging and a correlating surface property comparison was carried out by BET analysis. The Fourier-transform infrared spectra with attenuated total reflectance (FTIR–ATR) spectra were obtained by a Nicolet 6700 FTIR spectrometer (Thermo-Nicolet, USA) in conjunction with a GladiATR diamond ATR attachment (PIKE, USA). The FTIR-ATR spectra were measured in the spectral range of 4000–400 cm^−1^, resolution 4 cm^−1^, number of spectra accumulations 64, and Happ–Genzel apodization. The CI was calculated following the previously reported method (Eq. (2))^61^. The thermal decomposition property was characterized by thermogravimetric analysis (TGA) using a Setsys Evolution (Setaram, France). Elemental composition analysis was performed by SEM (MAIA3, Tescan, Czech Republic) coupled with an energy-dispersive X-ray detector (Oxford Instruments, UK) and equipped with a field emission gun and cryogenic system PP3010 (Quorum Technologies Ltd., Sussex, UK).

## Supplementary information

Supplementary Information

Description of Additional Supplementary Files

Supplementary Movie 1

Supplementary Movie 2

Supplementary Movie 3

Supplementary Movie 4

Supplementary Movie 5

Supplementary Movie 6

Supplementary Movie 7

## Data Availability

The data generated in this study are provided in the paper or its Supplementary Information and FigShare repository (10.6084/m9.figshare.22045199).
