# Supplementary material for: Magnetically boosted 1D photoactive microswarm for COVID-19 face mask disruption
Source: Nat Commun. 2023 Feb 20;14:935. doi: 10.1038/s41467-023-36650-6 (PMC9939864; doi:10.1038/s41467-023-36650-6)
Supplement: Supplementary file 1 — Supplementary Information [file 41467_2023_36650_MOESM1_ESM.pdf]

Supplementary Information

Magnetically boosted 1D photoactive microswarm for COVID-19 face mask disruption

Jeonghyo Kim,[1] Carmen C. Mayorga-Martinez,[1] Martin Pumera[1,2,3,4,]*

[1]Center for Advanced Functional Nanorobots, Department of Inorganic Chemistry, Faculty of Chemical Technology, University of Chemistry and Technology Prague, Technická 5, 166 28 Prague, Czech Republic

[2]Faculty of Electrical Engineering and Computer Science, VSB - Technical University of Ostrava, 17. listopadu 2172/15, 70800 Ostrava, Czech Republic

[3]Department of Chemical and Biomolecular Engineering, Yonsei University, 50 Yonsei-ro, Seodaemun-gu, Seoul 03722, Korea

[4]Department of Medical Research, China Medical University Hospital, China Medical University, No. 91 Hsueh-Shih Road, Taichung, Taiwan

*Corresponding author: pumera.research@gmail.com

Supplementary Methods

Mass spectrometry analysis and cytotoxicity assay of the liquid sample after the photocatalytic treatment

The 1D microswarm-treated PP filter membranes were exposed to light illumination for 30 h, following the same procedure performed in the previous experiments. Subsequently, the resulting reaction solution was collected and used for by-product analysis and cytotoxicity assay. The analysis of by-products was performed with a TRACE GC Ultra gas chromatograph (Thermo Scientific, USA) coupled with a quadrupole mass spectrometer (ISQ, Thermo Scientific, USA). SPME equipment with 65 μm polydimethylsiloxane/divinylbenzene (PDMS/DVB) fiber (Supelco, USA) was used to extract and concentrate the volatiles from the samples. The chromatographic separation was achieved using a DB-5MS UI column (60 m × 0.32 mm, film 1 μm; J&W Agilent), and the flow rate of the carrier gas (helium 6.0, SIAD, Czech Republic) was 1.5 mL/min. The initial temperature of the column oven was 40°C (held for 5 min), then ramped at 15°C/min to 290°C. The ionization energy was 70 eV, and the temperature of the ion source was set to 230°C. For the cytotoxicity assay, HeLa cells (cervical adeno-carcinoma, ATCC, CCL-2TM) were cultured in an EMEM medium with 5% FBS at standard conditions (37°C, 5% CO_2). Cell viability was evaluated by resazurin assay (Alamar Blue) and the fluorescence was measured (ex./em. 560/590 nm) using a plate reader.

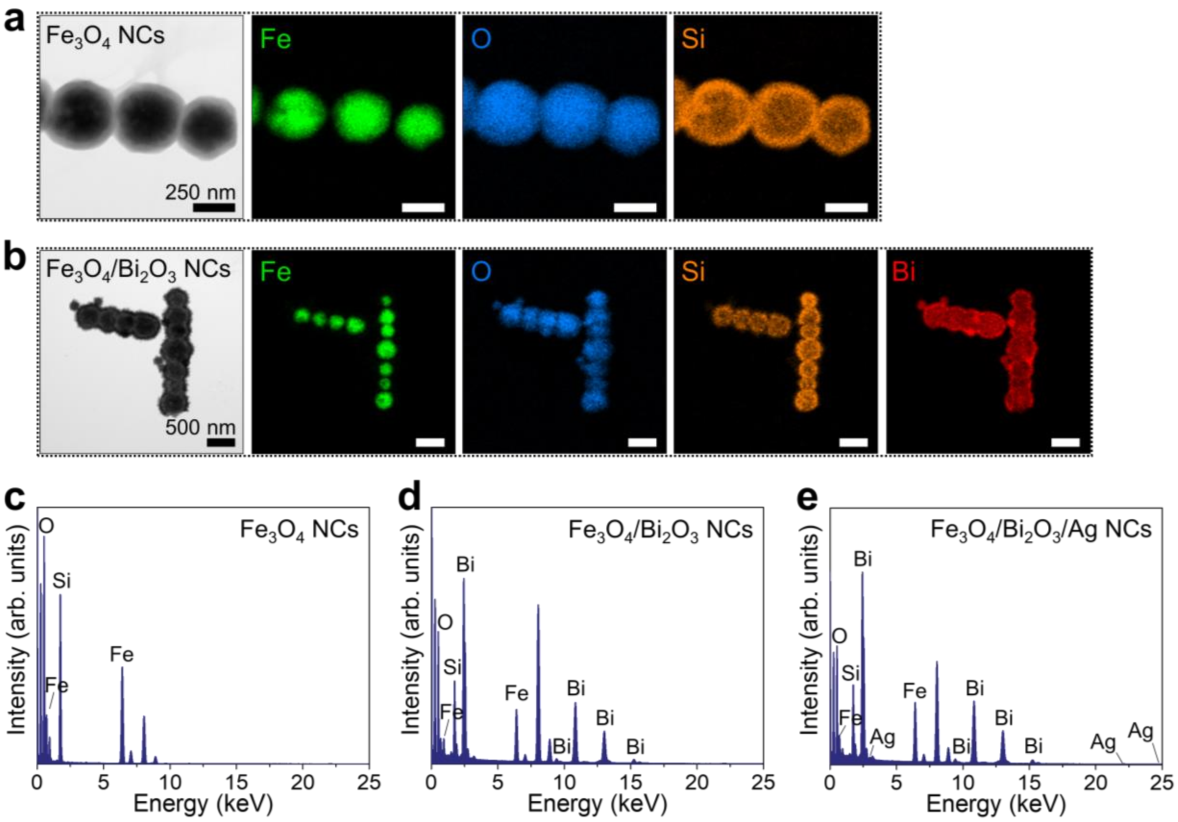

Supplementary Fig. 1. (a-b) TEM-EDX elemental mapping images of Fe_3O_4 NCs (a), and Fe_3O_4/Bi_2O_3 NCs (b). (c-e) EDX spectra of the Fe_3O_4 NCs (c), Fe_3O_4/Bi_2O_3 NCs (d), and $Fe_3O_4/Bi_2O_3/Ag$ NCs (e) corresponding to the TEM-EDX images shown in Supplementary Fig.1a, b and Fig. 2i.

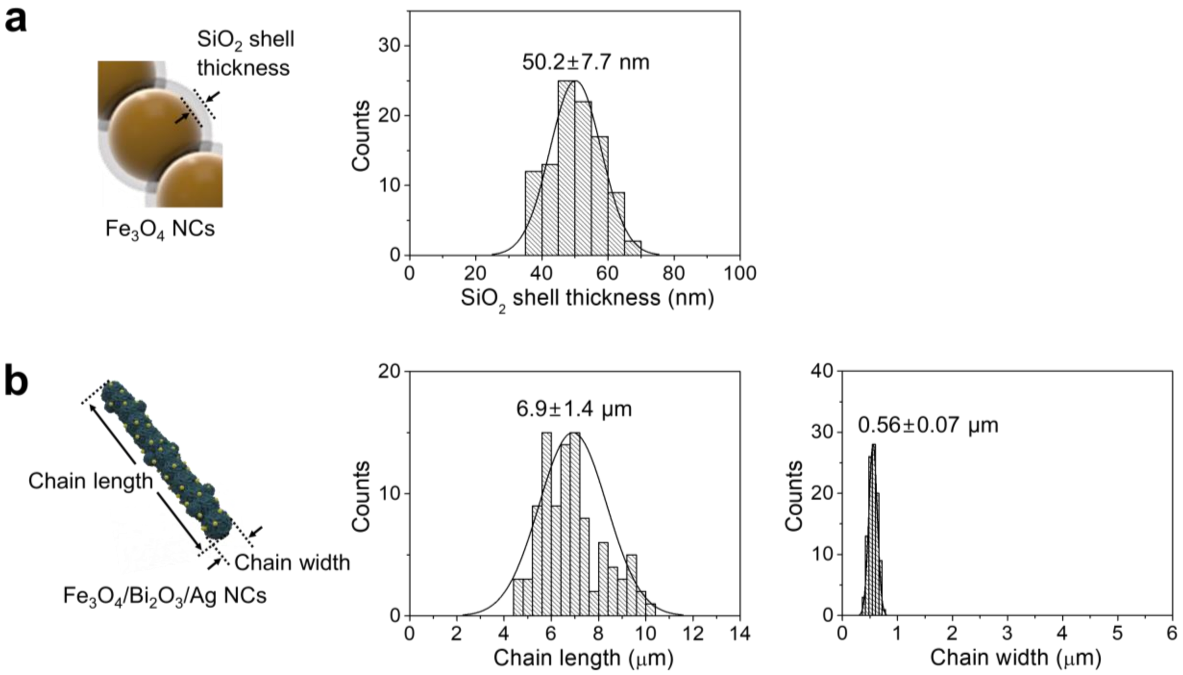

Supplementary Fig. 2. (a-b) SiO_2 shell thickness (a), and length and width (b) distribution histograms of the 1D magnetic photoactive microrobots.

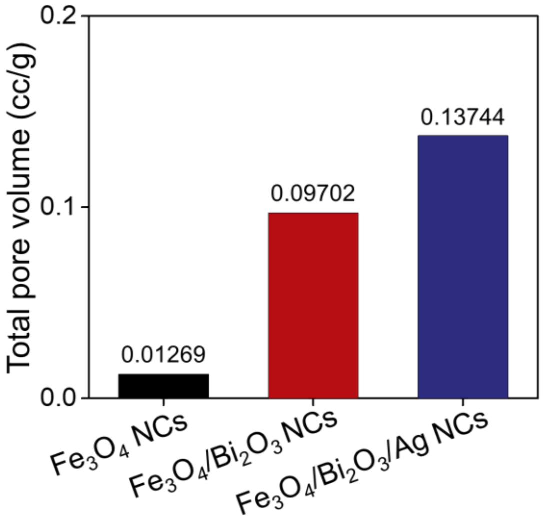

Supplementary Fig. 3. Comparison of BET total pore volumes for Fe_3O_4 NCs (black), Fe_3O_4/Bi_2O_3 NCs (red), and $Fe_3O_4/Bi_2O_3/Ag$ NCs (blue).

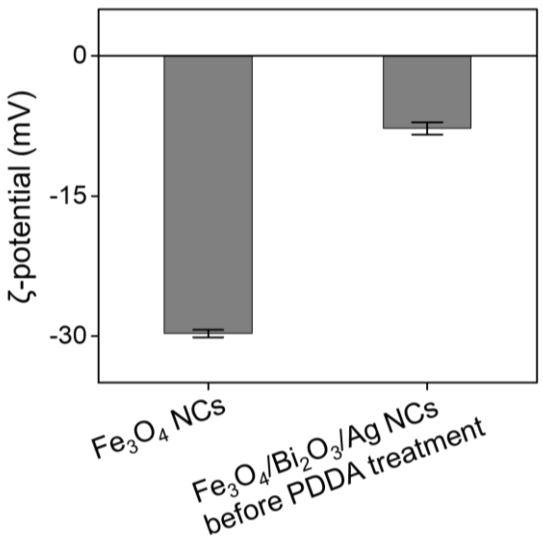

Supplementary Fig. 4. Zeta potentials of Fe_3O_4 NCs, and $Fe_3O_4/Bi_2O_3/Ag$ NCs before surface modification. Error bars indicate standard deviation from triplicate measurements.

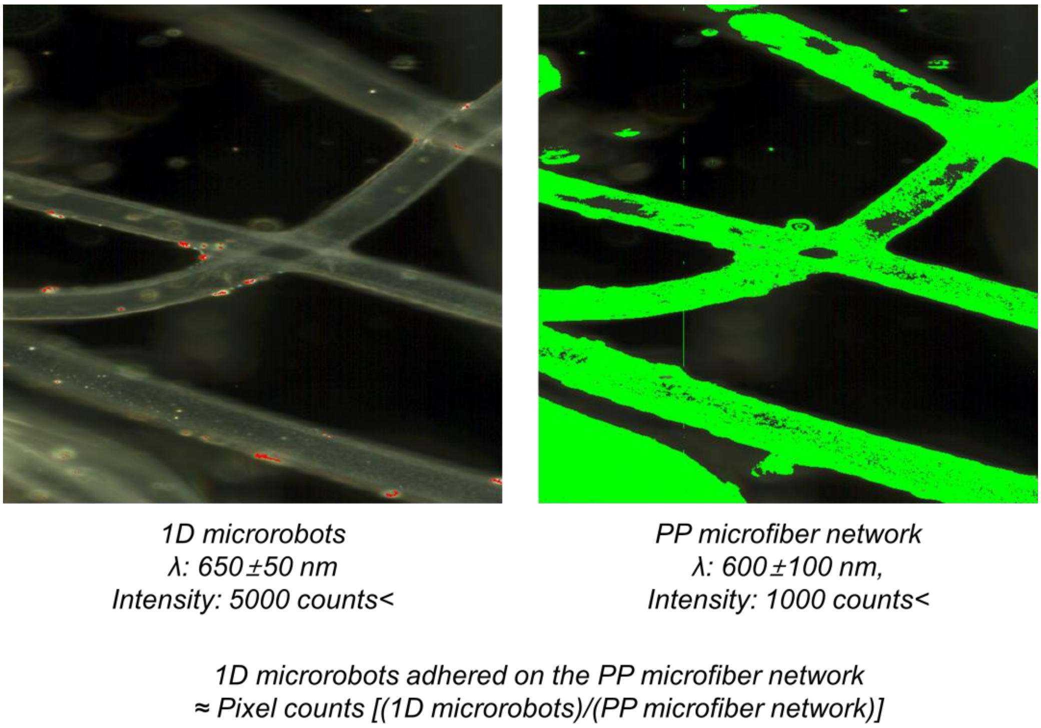

Supplementary Fig. 5. Enhanced dark-field hyperspectral mapping analysis of the adhered 1D microrobots and the PP microfiber network.

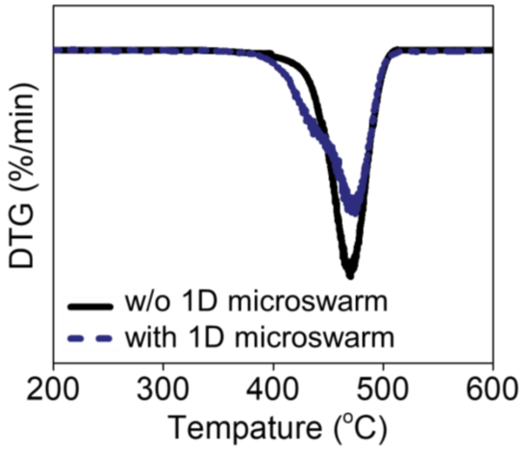

Supplementary Fig. 6. DTG curves of the polypropylene filter membranes after photoactivation with and without 1D microswarm treatment.

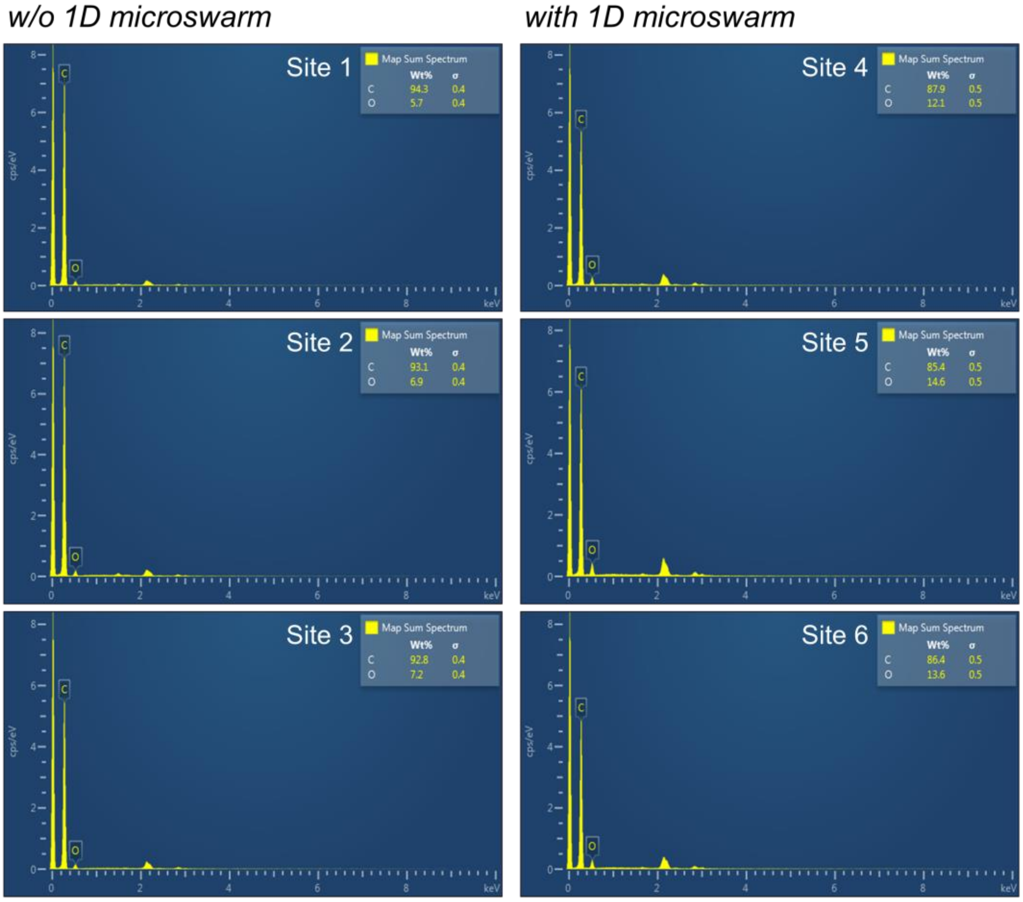

Supplementary Fig. 7. EDX spectra and elemental composition data corresponding to the SEM-EDX images shown in Fig. 7d.

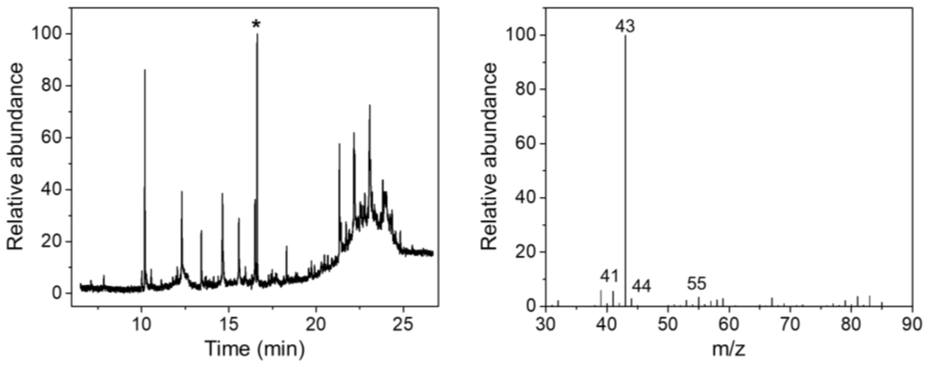

Supplementary Fig. 8. SPME-GC-MS analysis of the liquid sample resulting from the photocatalytic treatment of the PP filter membrane with 1D microrobots.

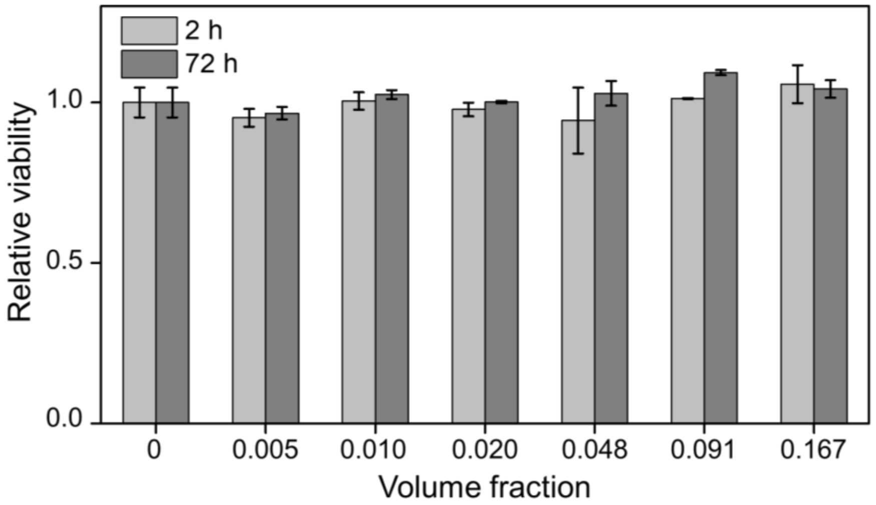

Supplementary Fig. 9. Cytotoxicity evaluation of the liquid sample after the photocatalytic treatment of the PP filter membrane with 1D microrobots. Cell viability assays were performed for the HeLa cell line after 2 h and 72 h incubation with the liquid sample. Data are presented as mean ± standard deviation of duplicate measurements.

Supplementary Table 1. Comparison of the different microrobot-based strategies for plastic waste treatment.

Microrobot material	Powering sources	Motion guidance	Target plastics	Treatment capability	Mechanism	Ref.
Au@Ni@TiO_2	UV-light (H_2O_2) Magnetic	n.s.	PS (particle), PE, etc (microplastics from personal care products and environment)	Removal	Phoretic interactions/ shoveling	1
Sunflower pollen grains	Magnetic	Magnetic	PS (particle)	Removal	Noncontact shoveling	2
$Ti_3C_2T_x$-γ-Fe_2O_3/Pt/TiO_2	UV-light Magnetic	n.s.	PS (particle)	Removal	Electrostatic interaction	3
Ion-exchange resin/Fe_3O_4	Self-electrophoresis	Magnetic	PS, PVC, PMMA, etc (particle)	Removal	Long-range dynamic attraction and electrostatic adsorption	4
Bi_2WO_6	UV-light (H_2O_2)	n.s.	PP (commercial wet wipe)	Degradation	Photocatalysis	5
$BiVO_4$/Fe_3O_4	UV-light (H_2O_2) Magnetic	Magnetic	PLA, PCL, PET, PP (plastic film)	Degradation Removal	Photocatalysis Adhesion	6
PDA@Fe_3O_4/Lipase	Magnetic	Magnetic	PCL (microplastic)	Degradation Removal	Enzymatic Adhesion	7
Fe_3O_4/Bi_2O_3/Ag	Magnetic	Magnetic	PP (face mask)	Degradation Removal	Photocatalysis Adhesion	*This study*

Abbreviations: n.s., not specified; PS, polystyrene; PE, polyethylene; PVC, polyvinyl chloride; PMMA, poly(methyl methacrylate); PP, polypropylene; PLA, polylactic acid; PCL, polycaprolactone, PET, polyethylene terephthalate.

Supplementary References

1. Wang, L., Kaeppler, A., Fischer, D., Simmchen, J. Photocatalytic TiO_2 micromotors for removal of microplastics and suspended matter. *ACS Applied Materials & Interfaces* 11, 32937-32944 (2019).

2. Sun, M., Chen, W., Fan, X., Tian, C., Sun, L., Xie, H. Cooperative recyclable magnetic microsubmarines for oil and microplastics removal from water. *Applied Materials Today* 20, 100682 (2020).

3. Urso, M., Ussia, M., Novotný, F., Pumera, M. Trapping and detecting nanoplastics by MXene-derived oxide microrobots. *Nature Communications* 13, 1-14 (2022).

4. Li, W. et al. Self-driven magnetorobots for recyclable and scalable micro/nanoplastic removal from nonmarine waters. *Science Advances* 8, eade1731 (2022).

5. Villa, K., Děkanovský, L., Plutnar, J., Kosina, J., Pumera, M. Swarming of perovskite-like Bi_2WO_6 microrobots destroy textile fibers under visible light. *Advanced Functional Materials* 30, 2007073 (2020).

6. Beladi-Mousavi, S. M., Hermanova, S., Ying, Y., Plutnar, J., Pumera, M. A maze in plastic wastes: autonomous motile photocatalytic microrobots against microplastics. *ACS Applied Materials & Interfaces* 13, 25102-25110 (2021).

7. Zhou, H., Mayorga-Martinez, C. C., Pumera, M. Microplastic removal and degradation by mussel-inspired adhesive magnetic/enzymatic microrobots. *Small Methods* 5, 2100230 (2021).
