# Supplementary material for: Magnetically boosted 1D photoactive microswarm for COVID-19 face mask disruption
Source: Nat Commun. 2023 Feb 20;14:935. doi: 10.1038/s41467-023-36650-6 (PMC9939864; doi:10.1038/s41467-023-36650-6)
Supplement: Supplementary file 2 — Description of Additional Supplementary Files [file 41467_2023_36650_MOESM2_ESM.pdf]

Description of Additional Supplementary Files

File Name: Supplementary Movie 1
Description: Magneticpowered propulsion of the 1D magnetic photoactive microrobots at different speed.

File Name: Supplementary Movie 2
Description: Steerable locomotion in the manual navigation mode.

File Name: Supplementary Movie 3
Description: Programmed locomotion in the automated navigation mode.

File Name: Supplementary Movie 4
Description: Collective motion of magnetically boosted 1D microswarm.

File Name: Supplementary Movie 5
Description: Fish schoolinglike 1D microswarm and active interaction with microfiber network.

File Name: Supplementary Movie 6
Description: Comparison of microfiber network interaction with static 1D microrobots.

File Name: Supplementary Movie 7
Description: Magnetic separation of 1D microswarm-treated PP fiber membrane and microdebris.
